# Pulmonary Alveolar Microlithiasis and Rheumatoid Arthritis: A Case Report and Review of the Literature

**DOI:** 10.1155/2021/8811507

**Published:** 2021-05-31

**Authors:** Waleed Hafiz, Ahmedhusam Alahmed, Mohammed Alahmadi, Rakan Alotaibi, Abdullah Alsharif, Safwan Alim, Mohammed Mokhtar, Kholoud Al-Maabdi, Omaima Badr

**Affiliations:** ^1^Department of Medicine, College of Medicine, Umm Al-Qura University, Makkah, Saudi Arabia; ^2^Department of Medicine, Alnoor Specialist Hospital, Makkah, Saudi Arabia; ^3^Department of Medicine, East Jeddah Hospital, Jeddah, Saudi Arabia

## Abstract

Pulmonary alveolar microlithiasis is a rare autosomal recessive condition that is characterized by the formation of excessive calcium phosphate microliths in the alveoli. Most patients are diagnosed in adulthood due to the slow progression of the disease. Children with this disease are asymptomatic, and changes in the lung parenchyma are usually discovered incidentally. The diagnosis is made by the combination of a positive chest imaging and histological examination. Rheumatoid arthritis (RA) is a chronic systemic autoimmune disease characterized by chronic seropositive symmetrical inflammatory polyarthritis with numerous extra-articular manifestations. It targets the lining of the synovial membranes, frequently affects females more than males, and is treated with the disease-modifying antirheumatic drugs (DMARDs). If left untreated, it leads to increased morbidity, mortality, and socioeconomic burdens. In this case, we report a 19-year-old young man who presented with clinical and radiographic features of PAM associated with RA.

## 1. Introduction

Pulmonary alveolar microlithiasis (PAM) is a fairly rare lung disease which is characterized by the deposition of alveolar calcium and phosphate in the lung tissues bilaterally and predominantly affecting the lower and middle lung zones. The etiology and pathogenesis are not completely understood [1, 2].

PAM is a genetic disease caused by a mutation in the SLC34A2 gene. This gene is a sodium-dependent phosphate transporter expressed in the lungs which is essential in the removal of phospholipids from the alveolar spaces. Dysfunctional SLC34A2 leads to the accumulation of calcium and phosphate causing microlithiasis [3].

PAM is considered an autosomal recessive disorder and is associated with consanguinity. A majority of patients have at least one affected sibling. Over 1200 cases were reported in the literature, and its prevalence is unknown. 35.8% of PAM patients are diagnosed before the age of 20 and 88.2% before the age of 55 [4].

In early stages, patients are asymptomatic with a usual slow progression. Over time, they develop exertional dyspnea, dry cough, weakness, chest pain, cyanosis, hemoptysis, and pneumothorax. Physical examination may show rales and bilateral clubbing of the finger. Additionally, it has been shown that smoking and infection may hasten the disease progress [4].

PAM is mainly diagnosed by imaging with the support of clinical manifestations. The typical findings of PAM on a plain radiograph are a fine scattered micronodular pattern forming a characteristic “sandstorm” sign. The borders of the heart, diaphragm, and vascular markings are obscured due to the dense calcification. Findings on the high-resolution computed tomography (HRCT) scan of the chest are classified into several stages based on the severity of imaging abnormalities. These range from the precalcific phase and calcific micronodules to ground-glass opacity, thickening of the intralobular septa, and the classic white lung sign [3].

Lung biopsy shows classical histopathologic features of PAM which are diffuse filling of alveolar air spaces by calcospherites, lamellated calcifications, fibrosis, and opacification in the late stages [3].

Unfortunately, the role of medical therapies such as systemic corticosteroids, calcium chelating agents, and bronchopulmonary lavage has been ineffective in slowing the disease progression. Diphosphonate therapy usage is still controversial and needs further evidence [5]. The only effective management is lung transplant which has shown to increase patient's survival for up to 15 years without recurrence [6].

Prognosis of PAM remains unclear, but according to a Japanese study which included 53 patients showed that 34.1–42.9 % died within 10–49 years after the diagnosis, most commonly due to respiratory failure [4].

Rheumatoid arthritis (RA) is a systemic autoimmune inflammatory disease that most commonly affects the joints causing progressive, symmetric, and erosive destruction of the cartilage and bone. It is usually associated with autoantibody production such as rheumatoid factor (RF) and antibodies against citrullinated peptides (anti-CCP). RA affects ∼1% of the population in developed countries. Although joint disease is the main presentation, there are a number of extra-articular manifestations including lung disease, cardiovascular disease, subcutaneous nodule formation, vasculitis, and inflammatory eye disease. Of these manifestations, lung disease is a major contributor to morbidity. In some cases, respiratory symptoms may precede articular symptoms [7].

In this report, we present a case of PAM with associated preclinical RA.

## 2. Case Presentation

A 19-year-old young man, not known to have any medical illness, presented to the pulmonology clinic for the first time in November 2019 with progressive difficulty in breathing and dry cough for 8 months. He also noted pain in multiple metacarpophalangeal (MCP) and proximal interphalangeal (PIP) joints bilaterally as well as the right wrist and both knees. This pain was not associated with swelling; however, he admitted daily morning stiffness that lasts for up to 30 minutes and improves with activity. The family history was notable for a sister with confirmed diagnosis of PAM. On his initial presentation, the patient was vitally stable with an oxygen saturation of 93% on room air. Physical examination revealed digital clubbing, symmetrical reduction in the chest expansion, bilateral dull percussion note, especially over the lower part of the chest, and vesicular breath sounds with bilateral basal inspiratory crepitation. He had multiple tender MCP and PIP joints, right wrist, and both knees with no evidence of joint swelling, effusion, or deformity. Cardiac, abdominal, neurologic, and dermatologic examinations were unremarkable.

Laboratory investigations revealed the following results: WBC 7.92 × 10^9/L (normal range: 4–11 × 10^9/L), hemoglobin 120.00 g/L (normal: 130–170 g/L), MCV 82.5 FI (normal range: 83–101 FI), platelets 431 × 10^9/L (normal range: 150–400 × 10^9/L), CRP 7.28 mg/dl (normal range: 0–0.3 mg/dl), ESR 105 mm/h (normal range: 3–10 mm/h), rheumatoid factor 8060 IU/mL (normal range: 0–15.9 IU/mL), positive anti-CCP and antinuclear antibodies, negative anti-ds DNA, P-ANCA, and C-ANCA, and normal complements.

**Figure 1:**
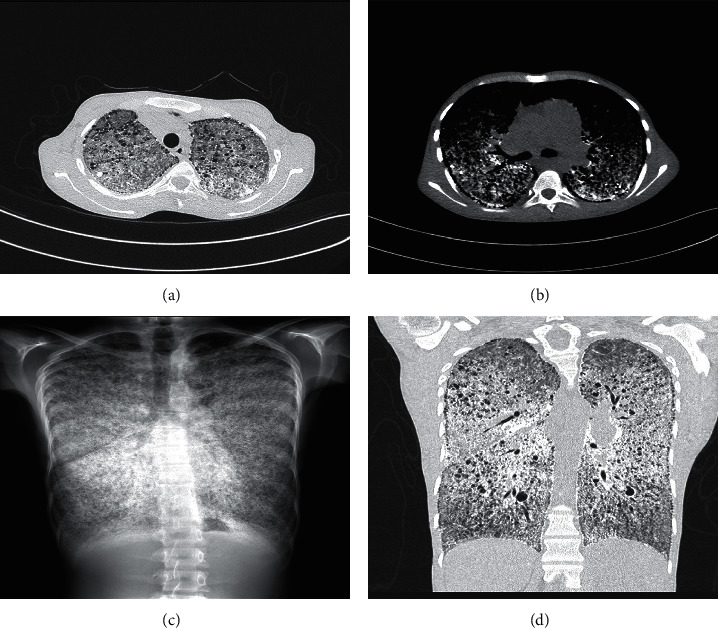
Multiple nodules of calcium density with an alveolar distribution affecting all lobes, septal and reticular thickening, and subpleural cysts.

The patient was admitted under the pulmonary medicine team for further assessment. Sputum culture tested negative for microorganisms, including acid-fast bacilli. The high-resolution computed tomography (HRCT) scan of the chest showed bilateral macronodular shadows with basal calcification, sand-like calcification, and black pleura sign. These findings were consistent with advanced PAM (Figure 1).

Although histopathology is usually required to confirm the diagnosis, the high clinical suspicion along with strong family history and classical radiologic findings were all thought to be sufficient to diagnose this patient with PAM. He was started on systemic steroids and oxygen therapy and was referred to thoracic surgery to initiate lung transplant workup.

The patient was also seen by the rheumatology team for assessment of his polyarthralgia. In the light of absent symptoms and signs of inflammatory arthritis but strongly positive serology, he was diagnosed with preclinical RA and was started on hydroxychloroquine therapy to halt/delay the progression to clinical RA.

**Figure 2:**
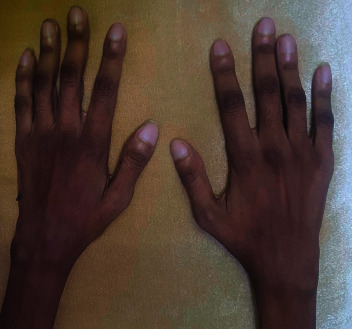
A picture of the patient's hands showing nail clubbing and swelling of several PIP and MCP joints.

A few months later, while waiting for lung transplant, he presented to the rheumatology clinic for follow-up. He admitted noncompliance to hydroxychloroquine and prednisolone. He had flare of his joint symptoms with multiple tender and swollen joints (Figure 2). Bedside musculoskeletal ultrasound of the hands revealed grade 2 synovitis of several MCP and PIP joints bilaterally. Based on this, he was diagnosed with seropositive RA, and he was restarted on his treatment.

## 3. Discussion

A variety of lung manifestations are known to develop in patients with RA. These include pulmonary parenchymal disease, pleurisy, airway disease, and pulmonary vascular disease [7]. The pathoetiology by which lung diseases occur in RA is not well understood. Several mechanisms have been postulated, such as the presence of autoantibodies and local production of inflammatory cytokines, impaired fluid resorption in the inflamed pleura, and inflammation of the airways and pulmonary vasculature [7, 8].

Other than the aforementioned lung manifestations, the literature review supports possible association between PAM and RA [4]. Three cases of PAM-associated RA had been described. The first case was reported in India and described a 30-year-old man with RA diagnosed one year prior to presenting with cough and whitish scanty sputum. Chest X-ray showed typical signs of PAM, and HRCT confirmed the diagnosis [9]. The second case from Colombia was a 44-year-old man who had RA for 15 years before presenting with epigastric pain, nausea, undocumented fever, and positive history of the first-degree relative with the diagnosis PAM. During his workup, the diagnosis of PAM was confirmed by chest X-ray and lung biopsy [10]. The third case was reported in Turkey and described a 17-year-old girl who presented with an 8-month history of pain and swelling in her knees, ankles, and hands. She was diagnosed with RA. Chest X-ray raised the suspicion of the incidental finding of PAM which was later confirmed with HRCT and lung biopsy [11].  Table 1 provides a summary of all cases of PAM-associated RA that are reported in the literature.

Up to our knowledge, our case is the fourth to be reported in the literature. Unlike the previous reported cases, it does not only confirm the association between PAM and RA but also shows that PAM may predate the onset of RA.

## 4. Conclusion

PAM is a rare lung disease in patients with RA. The etiopathologic link between PAM and RA is interesting yet unclear. We support the theory suggested by the authors who reported the previous cases that PAM can induce an autoimmune process that may initiate the pathogenesis of RA. We think that more cases of PAM-associated RA are needed to further explore this link.

## Figures and Tables

**Table 1:** Summary of our case and all cases of PAM-associated RA reported in the literature.

	Our case	Case 1 [9]	Case 2 [10]	Case 3 [11]
Clinical data	A 19-year-old Saudi young man, presented with progressive shortness of breath, dry cough, polyarthralgia for 8 months, and a positive family history of the first-degree relative with PAM. His polyarthralgia progressed to inflammatory polyarthritis.	A 30-year-old Indian man with RA, presented with cough and whitish scanty sputum for one year.	A 44-year-old Colombian man with long-standing RA, presented with epigastric pain, nausea, undocumented fever, and a positive family history of the first-degree relative with PAM.	A 17-year-old Turkish young woman, presented with a history of pain and swelling of the bilateral knees, ankles, and hands for 8 months.
Method of diagnosis	Clinical findings, classical PAM findings on HRCT, and positive RF and anti-CCP.	Clinical findings and radiological features of PAM on chest X-ray and HRCT.	Suspected diagnosis of PAM on chest X-ray, followed by lung biopsy which confirmed the diagnosis.	Elevated RF and suspected diagnosis of PAM on chest X-ray which was confirmed with HRCT and lung biopsy.
Duration between PAM and RA diagnosis	Preclinical RA was diagnosed one month after the diagnosis of PAM, which then progressed to RA in few months.	RA was diagnosed one year prior to the diagnosis of PAM.	RA was diagnosed 15 years prior to the onset of PAM features.	Both RA and PAM were diagnosed simultaneously.
Treatment	Home oxygen therapy, referral for lung transplantation, hydroxychloroquine, and prednisolone. Regular follow-up at the rheumatology clinic.	Referral for lung transplantation. Hydroxychloroquine and methotrexate to treat RA.	Oxygen therapy and regular follow-up at the pulmonary and rheumatology clinics.	Antirheumatic therapy (prednisolone, indomethacin, and salazopyrine) and regular follow-up at rheumatology and pulmonary clinics.
